# Dynamic damage evolution mechanism of buried cast iron pipeline under corrosion-blast coupling effects

**DOI:** 10.1038/s41598-026-54155-2

**Published:** 2026-05-26

**Authors:** Xia Yuqing, Jiang Nan, Yao Yingkang, Liang Zhenxing, Wu Tingyao

**Affiliations:** 1 State Key Laboratory of Precision Blasting, Jianghan University, Wuhan, 430056 China; 2 Hubei Key Laboratory of Blasting Engineering, Jianghan University, Wuhan, 430056 China; 3 School of Civil Engineering, Chongqing University, Chongqing, 400045 China; 4 Chongqing City Vocational College, Chongqing, 402160 China

**Keywords:** Old cast iron pipeline, Blasting vibration, Dynamic response, Corrosion defect, Failure mechanism, Pipe-soil interaction, Safety control, Engineering, Materials science

## Abstract

This study systematically investigated the dynamic response and failure mechanism of aging cast iron pipes affected by blasting operations in urban renewal projects. In response to key challenges such as the vibration safety risks brought by adjacent blasting activities, the lack of targeted control standards in the current “Blasting Safety Regulations”, and the insufficient understanding of the corrosion-blast coupling effects in existing research, a case study was conducted using the Beijing Metro Line 16 tunnel. Nine full-scale on-site blasting tests were carried out. The results showed that the vibration and strain propagation patterns were significantly different. When the explosion source was directly aimed at the lower part of the pipe, the blasting hazard was the greatest. The experiment also proved that the blasting exposure side was mainly subjected to axial tensile failure, which challenged the traditional engineering assumption. The study also developed and verified a refined numerical model including bending corrosion defects, quantifying the influence of corrosion depth on the dynamic response. The research results indicated that corrosion defects would cause significant stress concentration, and the peak vibration velocity and effective stress would increase sharply with the increase in corrosion depth. At a corrosion depth of 8 mm, the maximum effective stress during pipe blasting was 253% higher than that without corrosion. The corrosion depth is the core factor affecting the blasting safety threshold of cast iron pipes. The threshold decreased by 85.4% at 8 mm corrosion, and 4 mm is already close to the critical failure point. The current regulations have both overly strict requirements for non-corroded pipes and overly lenient requirements for old and corroded pipes. The arc-shaped defect model constructed in this study is more accurate, overturning the conventional understanding of control on the facing side of the explosion, and the proposed single-section explosive dosage control table can be directly applied, balancing safety and construction efficiency, providing a scientific basis for regulation revision and engineering control.

## Introduction

Underground pipelines serve as the core infrastructure for transporting energy, water resources, and information, forming an indispensable foundation for modern urban operations^1,2^. During the early stages of urban pipeline construction in China in the last century, cast iron pipes were widely adopted for water supply, gas distribution, and drainage systems due to their readily available raw materials, mature manufacturing processes, and cost-effectiveness, becoming the critical “underground arteries” of urban infrastructure networks^3^. Statistics indicate that cast iron pipes (including gray iron pipes and early ductile iron pipes) accounted for over 20% of urban water supply networks built before 2000, with some older districts exceeding 50%^4^. However, as urbanization enters a phase of existing infrastructure renewal, these decades-old pipelines face severe aging challenges^5,6^: Compared to steel pipes, PE pipes, and concrete pipes, cast iron pipes exhibit inherent brittleness, poor impact resistance, and seismic stability, leading to significantly increased accident rates under combined internal corrosion and external disturbances^7^. With ongoing urban renewal initiatives and underground space development, blasting excavation remains the primary method for hard rock excavation due to its efficiency and speed, widely applied in subway tunnels, deep foundation pits, and utility tunnels. Blasting vibrations, as a major hazardous effect, pose significant threats to adjacent underground pipelines^8^. Seismic waves generated by blasting propagate through soil-rock media, inducing ground vibrations and displacements around pipelines. These forces create instantaneous dynamic loads at the pipe-soil interface, potentially causing structural failure when exceeding the pipeline’s load-bearing capacity. For aging corroded cast iron pipelines, irregular corrosion defects compromise structural integrity. The dynamic stresses induced by blasting vibrations concentrate at defect locations, significantly increasing the risk of catastrophic failure^9^. Moreover, the repetitive loading characteristics of blasting vibrations lead to fatigue accumulation at corrosion sites, progressively deteriorating material properties. This ultimately results in pipe rupture, joint displacement, fluid leakage, and even road collapse, severely jeopardizing urban operational safety. As China’s urbanization transitions toward high-quality development, the operational safety and modernization of underground pipeline networks have garnered heightened attention^10^.

The national “Implementation Plan for Aging Urban Gas Pipeline Renewal and Renovation (2022–2025)” stipulates that the replacement of gray cast iron pipelines must be largely completed by the end of 2025. However, a significant number of aging cast iron pipelines remain in service during the transition period^11,12^. The current “Blasting Safety Regulations” (GB 6722 − 2014) primarily establishes blasting vibration safety standards for civil buildings and tunnels, without considering the corrosion characteristics of aging cast iron pipelines. There are still no unified technical standards for critical parameters such as safe blasting distances, surface controlled vibration velocities, and maximum allowable explosive charges per Sect^13^.. Construction sites often rely on empirical approaches for safety control, frequently facing a dilemma: either overly conservative standards that compromise construction efficiency or inadequate controls that create safety hazards. Therefore, systematic research on dynamic response characteristics and failure mechanisms of aging cast iron pipelines under adjacent blasting conditions, revealing their failure patterns and proposing targeted safety control methods, holds significant engineering value and practical implications for guiding similar construction projects, ensuring underground pipeline network safety, and promoting high-quality development of urban underground infrastructure^14^. Corrosion remains the primary cause of buried pipeline failures. Scholars worldwide have conducted extensive systematic studies on pipeline corrosion characteristics, stress mechanisms, and failure patterns through theoretical analysis, experimental testing, and numerical simulations^15^. Current research on pipeline corrosion characteristics indicates that corrosion evolution is governed by multiple factors including soil environment, transported media, material properties, and operational conditions. Key influencing factors encompass soil physicochemical properties, microbial activity, internal pipeline pressure, corrosive media, and pipe material types, all of which significantly affect corrosion behavior, defect morphology, and propagation rates^16^. Corrosion can be classified by location into inner wall corrosion (primarily caused by transported media) and outer wall corrosion (mainly resulting from soil electrochemical reactions and cathodic protection system failure). Geometrically, corrosion patterns include uniform corrosion, pitting corrosion, localized corrosion, and corrosion pit clusters, with pitting and localized corrosion often causing pronounced stress concentration around defects that severely compromise pipeline load-bearing capacity^17^. Corrosion also leads to progressive mechanical property degradation—gray iron pipes exhibit exponential toughness loss after corrosion. Researchers have developed engineering-applicable corrosion rate prediction models to support performance degradation assessment^18^. Regarding mechanical behavior and failure mechanisms, studies confirm that buried corroded pipelines experience stress responses resulting from combined effects of corrosion degradation, external loads, and soil-pipe interactions. Localized corrosion directly weakens mechanical properties by reducing effective load-bearing cross-sections and intensifying stress concentration at defects. Corrosion depth remains the primary factor influencing mechanical responses, with defects significantly amplifying damage severity under dynamic loads. Coupled corrosion with other defects can substantially reduce pipeline fatigue life^19^. Pipeline failure modes are influenced by a combination of corrosion characteristics, load types, and material properties. Common failure modes for metal pipelines include buckling failure, tensile failure, crack propagation, and joint failure. Under composite loads, dual failure modes such as tensile-buckling failure are also prone to occur^20^. The dynamic response and failure modes of buried pipelines subjected to blast vibrations are affected by multiple factors including soil conditions, material properties, and structural configurations. Researchers worldwide primarily employ three methodologies: field testing, physical experiments, and numerical simulations. In terms of pipeline dynamic response characteristics, field testing and full-scale blast experiments serve as fundamental research foundations. Through field monitoring conducted in various blasting projects, scholars have elucidated the propagation and attenuation patterns of blast vibration waves, clarified the impact of key parameters such as charge quantity, blast center distance, and internal pipeline pressure on vibration responses, and completed full-scale blast tests for multiple pipeline materials. With advancements in computer technology, numerical simulation has become the mainstream research approach, with dynamic finite element software like ANSYS/LS-DYNA and ABAQUS widely utilized. Existing studies validate the feasibility of numerical methods in analyzing pipeline blast responses, systematically compare dynamic response differences under varying operating conditions, and reveal stress evolution characteristics of pipe-soil coupled systems under blast loads. Regarding pipeline dynamic failure mechanisms, current research focuses on failure processes under blast loads, conducting in-depth analyses from perspectives including stratum deformation, material damage evolution, and structural vibration characteristics^21^. Research indicates that stratum deformation induced by blasting causes axial bending deformation in pipelines, with joint areas serving as critical weak zones for crack initiation and failure, where tensile stress predominantly drives structural damage. Scholars have developed various pipeline failure criteria and safety assessment methods based on material yield principles and ultimate strain limits, preliminarily investigating the impact of corrosion defects on blast stress concentration^22^. However, systematic studies on blast construction near aging cast iron pipelines remain insufficient, with three key deficiencies: First, the coupling mechanism between corrosion and blast vibration lacks in-depth exploration, resulting in inadequate understanding of dynamic responses, stress evolution, and fatigue failure mechanisms under blast loads for corroded pipelines. Second, collaborative pipe-soil dynamic response models under corrosion conditions remain incomplete, with insufficient comprehension of how soil parameters affect blast vibration propagation and energy dissipation, making it challenging to accurately assess pipeline stress states. Third, specialized safety control standards for aging cast iron pipelines are lacking, as existing empirical approaches exhibit significant variability, failing to establish quantitative relationships between blast parameters, ground vibration, and pipeline failure, thereby compromising safety evaluation reliability. Current research indicates that the corrosion development and structural performance degradation of buried cast iron pipelines are a complex process involving multiple factors in coupling. Besides the key factors such as corrosion depth, defect morphology, and load type that have been analyzed, external conditions such as soil environment, medium characteristics, and construction disturbances also significantly affect the service performance of the pipelines.

From the perspective of environmental factors, soil pH value, salt content, moisture content, and redox potential directly control the rate of electrochemical reactions, and acidic soil and high-salt environments will significantly accelerate pipe wall corrosion; microbial corrosion (MIC), as a common corrosion form of underground pipelines, the metabolic products of sulfate-reducing bacteria will destroy the passivation film, forming local pits and accelerating crack initiation. In terms of geology and external disturbances, human interference such as mining activities, pit excavation, adjacent construction vibration, and ground loading will change the stress state and soil constraint conditions of the pipeline, intensifying the relative displacement and stress concentration at the pipe-soil interface. Moreover, fluctuations in pipeline internal pressure, deterioration of joint sealing, uneven geological settlement, and freeze-thaw cycles will gradually weaken the structural integrity and further increase the failure risk when coupled with corrosion defects.

Most current studies still focus on the analysis of single factors, and the overall understanding of the coupling of multiple factors, the interaction of environment-mechanics-corrosion, is still insufficient. In particular, there is a lack of research on the damage evolution under the combined action of blasting vibration and complex environmental factors.

In summary, the existing research has not clearly revealed the coupling mechanism of corrosion defects and transient blasting loads, and there is a lack of quantitative understanding of how corrosion changes local stress concentration and controls failure modes. In response to this key research gap, this paper conducts full-scale on-site blasting tests and refined numerical simulations to systematically study the dynamic damage evolution laws of buried cast iron pipelines under corrosion-impact coupling. The main original contributions are as follows:

It reveals the stress concentration characteristics and strain distribution laws of corroded cast iron pipelines under blasting loads, and clearly indicates that the corrosion depth is the core controlling factor for dynamic response and safety threshold; it is the first time that experiments have confirmed that the axial tension on the backblast side is the main failure mode, overturning the traditional engineering cognition of controlling at the frontblast surface, and clarifying the most dangerous section and the most unfavorable blasting position; it establishes a pipe-soil dynamic coupling model considering arc-shaped corrosion defects, correcting the idealization deviations of traditional simplified models, and improving the accuracy of stress concentration and dynamic transfer calculations; it constructs a blasting safety threshold system considering the coupling effect of corrosion, and proposes a single-section charge quantity control table that can be directly applied to engineering, compensating for the deficiency of current norms in the applicability of aged corrosion pipelines. This research result can provide scientific basis and technical support for the safe control of adjacent blasting construction of old cast iron pipelines in urban renewal and the revision of norms.

## Field test plan

### Project overview

**Fig. 1:**
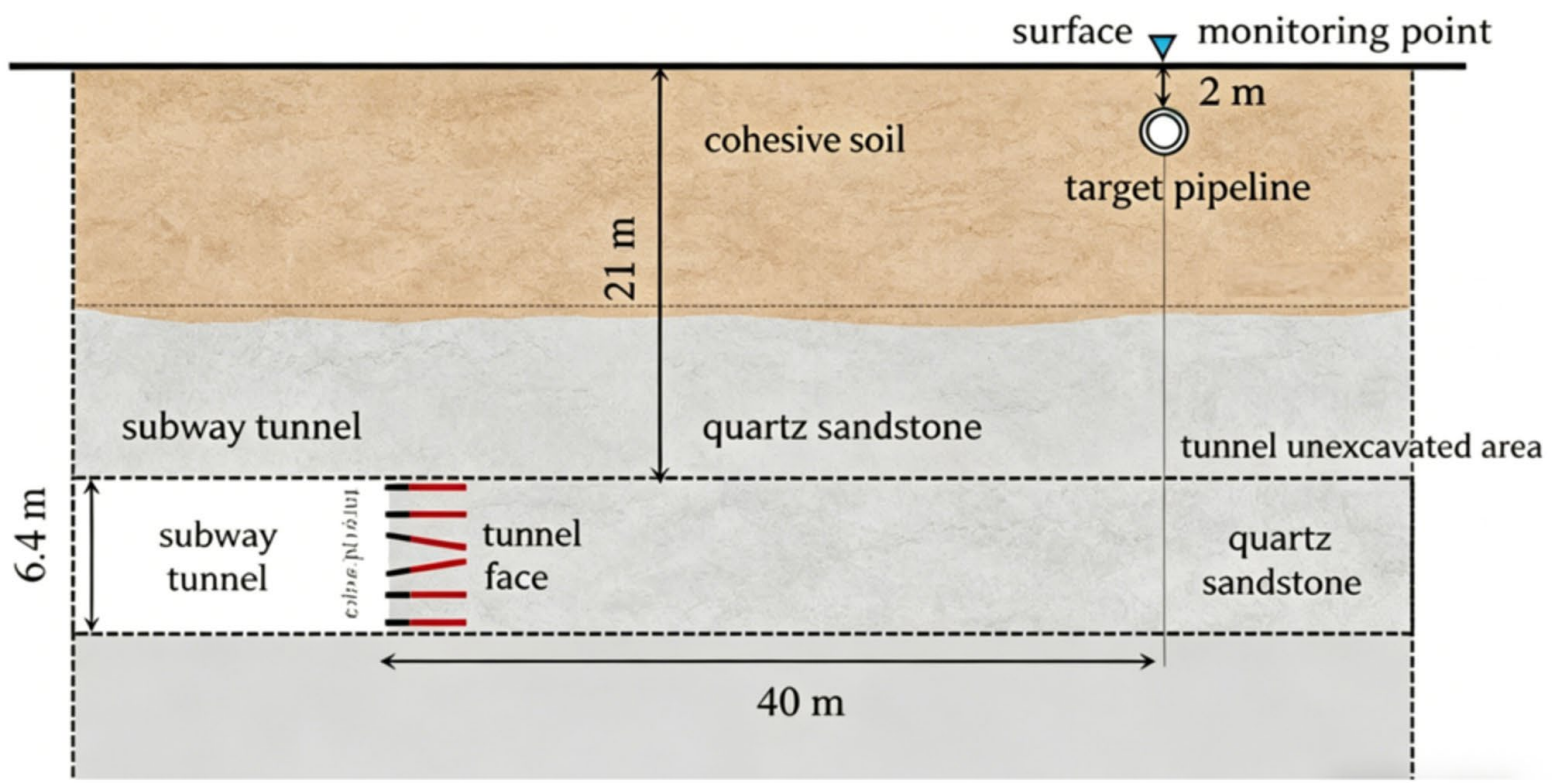
Schematic diagram of blasting engineering for cast iron pipeline crossing under Beijing Subway Line 16.

Beijing Subway Line 16 serves as the backbone rail transit line running north-south through the city. Starting from Wanpingcheng Station in Fengtai District, the line extends northward through Lize Financial Business District, Sanlihe Core Administrative Area, National Library, Suzhou Street, and Yongfeng Science Park before reaching Haidian Shanhou District, with its terminus at Beianhe Station. The entire 49.8-kilometer route is fully underground, featuring 29 stations. The study focuses on the Xibeiwang-Malianwa section, where the 920-meter-long tunnel adopts a horseshoe-shaped cross-section with a net height of 6.4 m, width of 6.3 m, and an arch burial depth of 21 m. The tunnel primarily traverses hard rock strata, utilizing drilling and blasting methods for excavation. Figure 1 illustrates the blasting engineering diagram for the cast iron pipeline crossing section of Beijing Subway Line 16.

Since field testing cannot directly assess buried pipelines through excavation exposure, this study conducts full-scale blasting experiments based on actual conditions from the northwest Wang to Ma Lian Wa station tunnel blasting excavation project of Beijing Subway Line 16. The research focuses on blast-induced dynamic responses during tunnel penetration through buried cast iron gas pipelines, systematically simulating construction loads from rock drilling and blasting methods in subway tunnel hard rock sections. The experiments capture vibration propagation patterns in cohesive soil layers and analyze vibration-strain response characteristics of buried cast iron pipelines.

### Trial protocol

This study focuses on the tunnel blasting excavation project for Beijing Subway Line 16’s Northwest Wang to Malianwa station section. Through systematic investigation of site geological conditions, pipeline types, and blasting monitoring data, it provides fundamental references for developing on-site test protocols. Based on these findings, a site leveling project in Beijing was ultimately selected as the blasting test location. The test site’s surface soil layer primarily consists of mixed fill soil and clay strata, with underlying bedrock composed of sandstone layers. Specific physical and mechanical parameters of each stratum are detailed in Table 1, while Table 2 presents parameters for pre-installed gas pipelines. Comparative analysis reveals that the silty clay layer exhibits characteristics closely resembling those of pipeline burial zones in Beijing’s urban core, demonstrating strong representativeness. Additionally, the underlying blasting rock strata show high similarity to excavation rock masses encountered in actual engineering projects.

**Table 1:** Geotechnical parameters of blasting site.

Stratum	Natural density γ/(kN·m3)	Angle of friction φ/(°)	Cohesion c/(kPa)	Bearing capacity fk/(kPa)
Banket	19.2	18	8	120 ~ 160
Silt clay	19.3	12	25	160 ~ 180
Malmstone	26.8	5.5	43	2000 ~ 4000

**Table 2:** Parameters of pre-installed gas pipelines.

Tubing	Elastic modulus E/(GPa)	Depth of burial h	Caliber d/(m)	Wall thickness δ/(m)	Poisson ratioµ
Cast iron	205	2	1	0.02	0.3

**Fig. 2:**
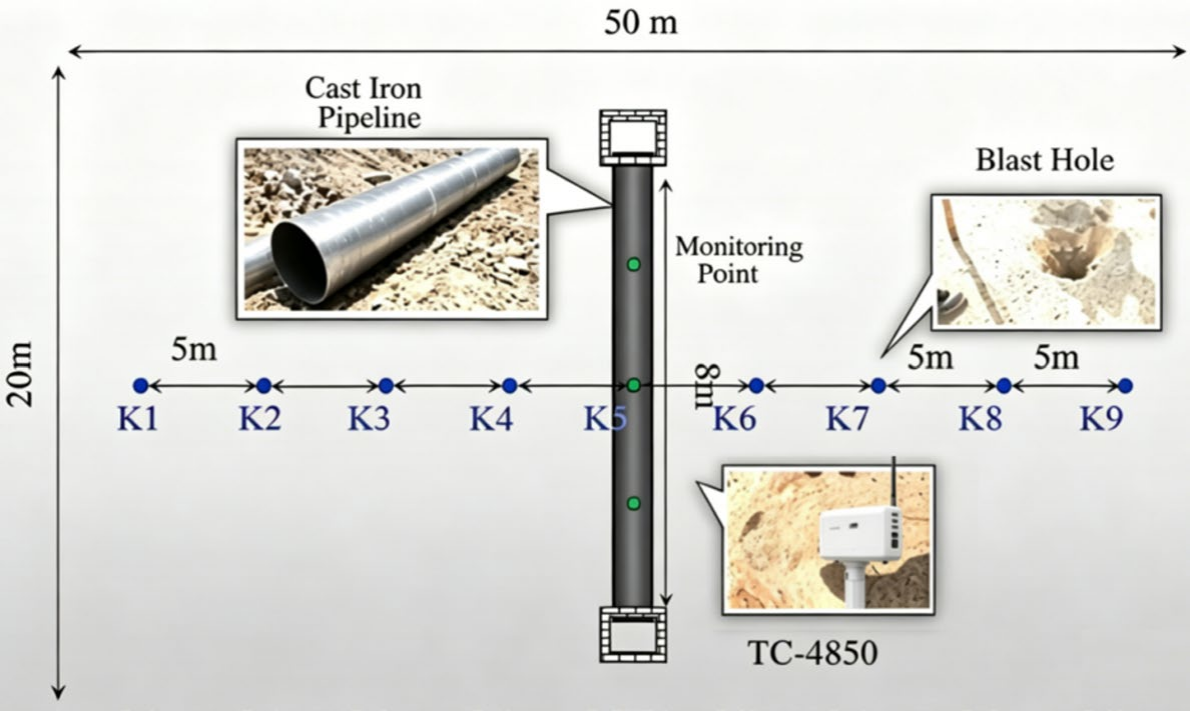
Schematic diagram of field experiment design.

Considering the engineering characteristics mentioned above, the blasting test was appropriately simplified to reduce implementation complexity while ensuring accurate load application, as illustrated in Fig. 2. To simulate the actual stress state of pipelines within soil media, the pipelines were embedded in a highly saturated silty clay layer at a depth of 2.0 m. The trenches were excavated using direct burial methods, with strict compaction control during backfilling to ensure tight pipe-soil contact. The pipelines had an axial length of 8 m—significantly exceeding their diameter—with reserved trench ends to establish free boundary conditions. These configurations effectively replicate real-world constraint conditions around the pipelines and at their ends, ensuring no additional pressure was applied during testing.

The blasting test employed No.2 rock emulsion explosives commonly used in engineering projects, with charge packages measuring 70 mm in diameter and 350 mm in length. To simulate the impact of different blast source positions on pipelines, nine sets of blast holes (K1-K9) were sequentially arranged along the pipeline axis from right to left, corresponding to operational condition codes 1–9. All blast holes were located vertically directly beneath the buried ductile iron pipeline and oriented downward. To accurately simulate the generation and propagation mechanisms of blasting loads, the maximum single‑stage charge weight used in practical construction was adopted as the simulation basis, with a charge weight of 9 kg per hole per stage. The blast holes were drilled to depths of 10 m and 8 m, a diameter of 80 mm, and a spacing of 5 m. Bottom concentrated charging was applied for all holes, and non‑electric detonators were used to initiate each hole individually by stage. This setup fully satisfied the requirements for seismic wave generation in actual blasting excavation operations.

**Fig. 3:**
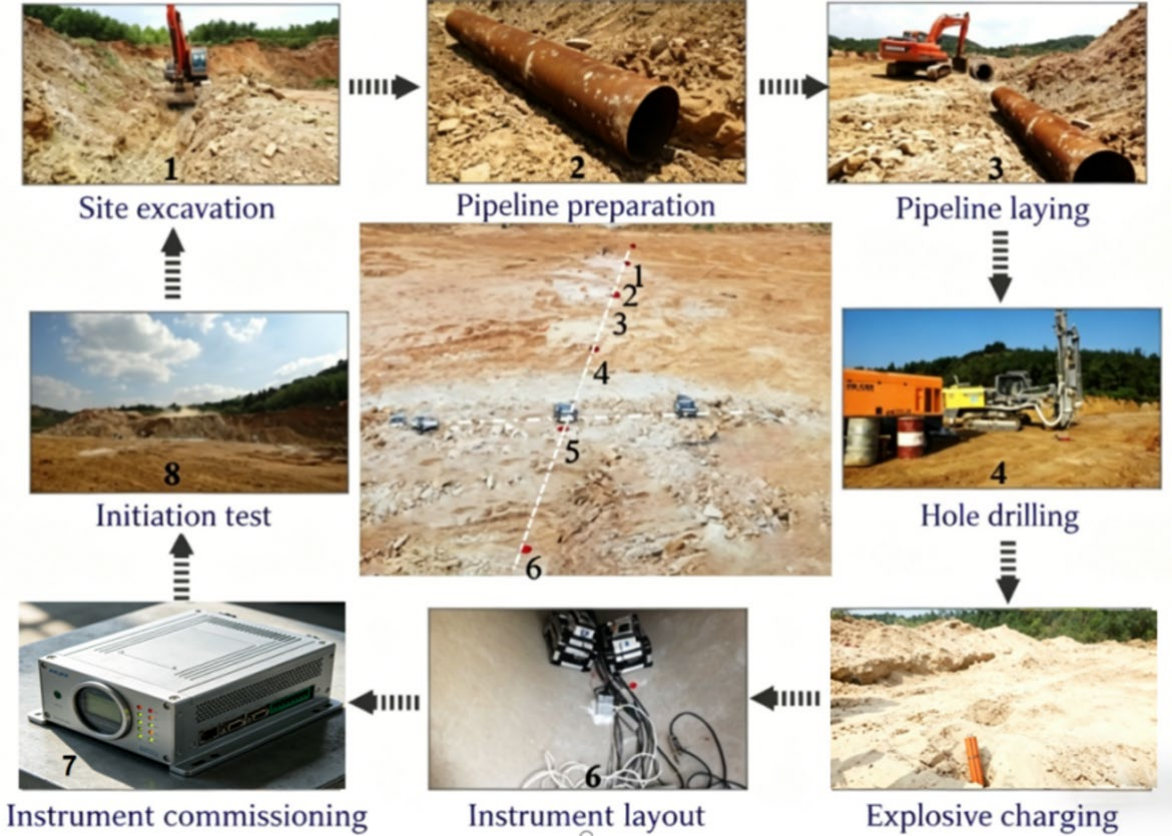
Implementation steps of field experiment.

Based on the blasting test design plan and monitoring layout described above, the main operational procedures for the field test are illustrated in Fig. 3.

1. Within the test site, a small excavator was initially employed to excavate a pre-designed trench with dimensions of 1.2 m × 1.2 m and a depth of 3 m to meet pipeline installation requirements. After trench formation, drilling operations were conducted using a φ90 mm Kaishan hydraulic drilling rig according to the borehole layout design plan.
2. Upon completion of drilling operations, dynamic strain gauges shall be sequentially attached to the inner pipe walls in accordance with measurement point layout requirements, accompanied by vibration velocity sensor installation and reserved connection port interfaces. The test pipe section shall then be hoisted into position and backfilled in layers until reaching the designed elevation through compaction. During backfilling, a portable compaction tester shall be employed for real-time monitoring of compaction density, ensuring it remains within the 90%−95% range to guarantee proper soil-pipe adhesion and structural stability.
3. After backfilling is completed, vibration velocity sensors are installed at predetermined surface locations. Subsequently, the charge loading and plugging procedures are performed sequentially according to the charge design, followed by connection of detonators and the initiation system. Data acquisition instruments are connected to each sensor and placed in a secure area for standby use.
4. Finally, detonate each blast hole in the predetermined sequence to conduct field tests and collect dynamic response data such as strain and vibration velocity. The interval between adjacent blast holes is 30 min. Prior to each detonation, the effectiveness of the previous blasting is inspected, and the instruments are calibrated.

This 9-group on-site test was conducted under the baseline conditions of no corrosion defects, a fixed burial depth of 2 m, and an internal pressure of 0 MPa. Only the horizontal relative position of the explosion source and the pipeline was changed to obtain the dynamic response laws of the pipeline under the most unfavorable blasting position. Due to the implementation conditions of the full-scale on-site test, the cost of pipeline prefabrication, and the safety control of blasting, this test did not carry out on-site measurements under different corrosion depths, soil cover thicknesses, and internal pressure conditions. To make up for the deficiencies in the on-site test conditions, this paper selected 3 typical corrosion defect conditions (corrosion depths of 4 mm, 6 mm, and 8 mm) as control conditions. Numerical simulations were carried out using the same material parameters and boundary conditions as the on-site tests, and the simulation results were compared and verified with the test data to form a complementary support of “baseline test + typical defect simulation”, thereby enhancing the coverage of the research conclusions.

This paper selected corrosion depths of 0, 2, 4, 6, and 8 mm as research parameters. The values were based on the following: (1) According to the on-site detection data of buried cast iron pipelines in Beijing’s old urban area, the corrosion depth of the outer wall of pipelines in service for more than 30 years is mostly within the range of 2–8 mm; (2) The wall thickness of the test pipeline is 20 mm, and 8 mm corresponds to a 40% reduction in wall thickness, which is a typical critical depth for brittle failure of cast iron pipelines; (3) Referring to relevant literature and standards on pipeline corrosion assessment at home and abroad, using 2 mm as an interval of uniform spacing can effectively characterize the entire process evolution law of corrosion defects from mild to severe, meeting the requirements of parameter sensitivity analysis and orthogonal experimental design.

### Test plan

**Fig. 4:**
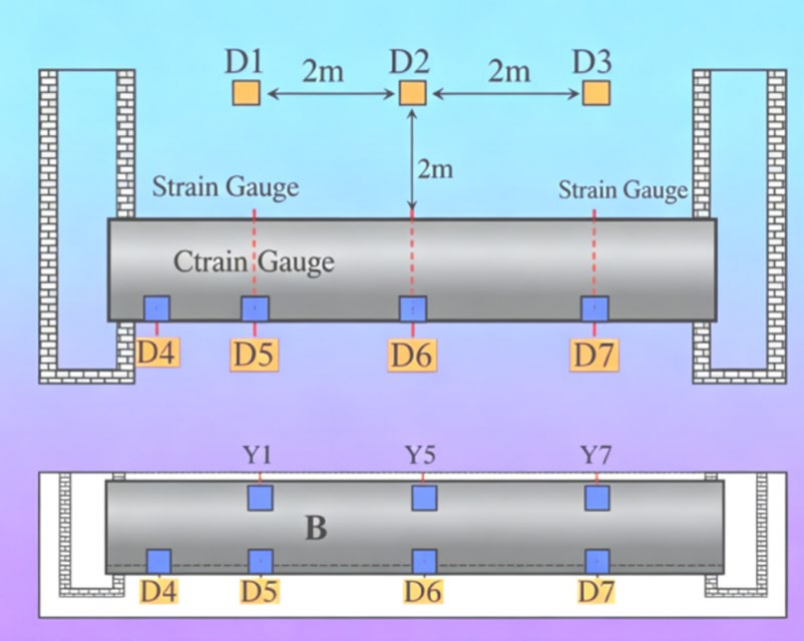
Schematic diagram of measurement point layout and testing instrument system for field experiments.

To systematically investigate the dynamic response characteristics of gas pipelines undergoing blasting operations, this experiment primarily collected three key response parameters: particle vibration velocity (vP) of the pipeline, surface vibration velocity (vG) above the pipeline, and dynamic strain (ε). Data acquisition was achieved through dynamic testing instruments deployed along the experimental pipeline section and surrounding geotechnical layers, enabling real-time monitoring during blasting processes to obtain accurate dynamic response data. The detailed layout of measurement points and test system configuration are illustrated in Fig. 4.

Vibration velocity data was collected using a TC-4850 blasting vibration meter, with measurement points installed inside the pipeline and directly above its surface. The specific locations included cross-section A at the pipeline’s midpoint and cross-section B 2.5 m from the pipeline edge, numbered sequentially as D1 to D7. Dynamic strain measurements were obtained through resistance strain gauges attached to the pipeline’s inner wall, featuring single-axis 80 mm gauge lengths and 120 Ω resistance values. Six measurement points were evenly distributed circumferentially at each monitoring section, with each point covering both circumferential and axial directions. Data was transmitted to the DH5956 dynamic data acquisition instrument via a 1/4 bridge configuration.

### Experimental analysis of dynamic response characteristics of cast iron pipelines

#### Vibration characteristics of pipe-soil systems

**Fig. 5:**
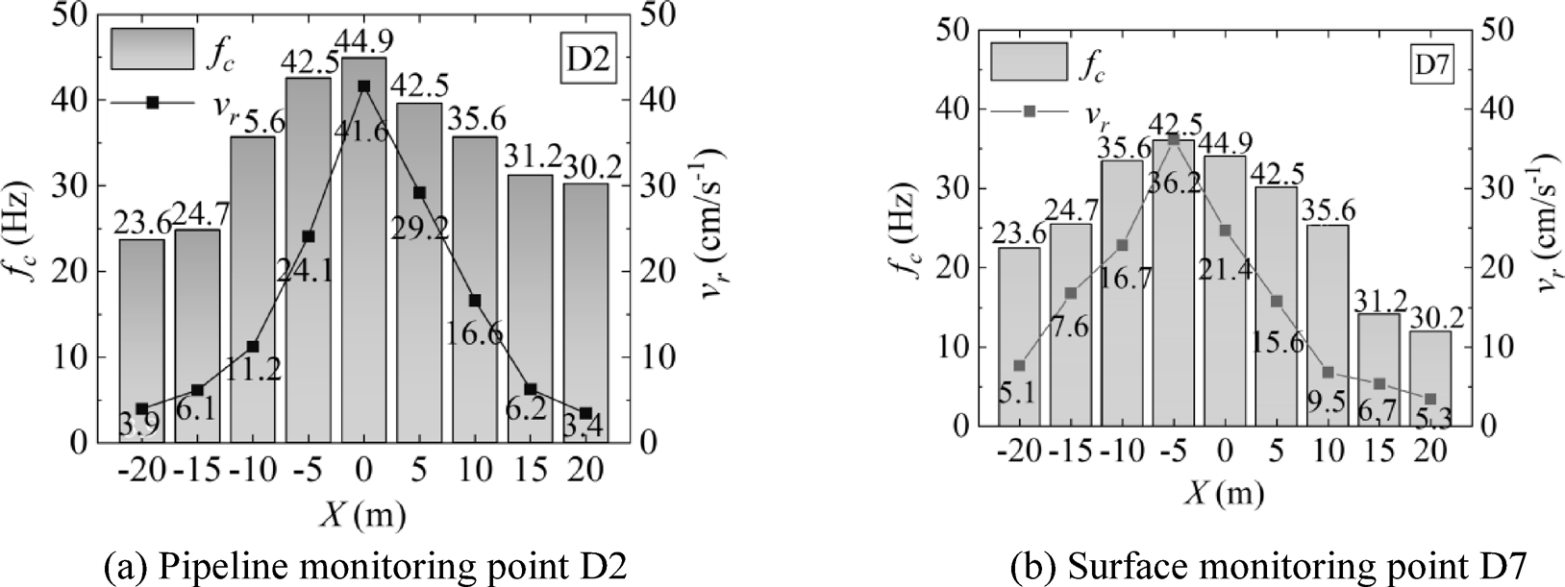
Statistical analysis of combined vibration velocities and maximum natural frequencies for pipelines and surface monitoring points.

In this experiment, nine rounds of blasting vibration data collection were completed with consistent explosive charges used across all tests, with only the relative position between the blast source and pipelines being altered. When the horizontal distance between the blast source center and pipeline axis reached 15 m, we analyzed the three-dimensional vibration velocity time-history curves recorded at surface monitoring point D7 and pipeline monitoring point D2. Frequency distribution analysis revealed that surface monitoring points exhibited predominant vibration frequencies concentrated between 10 Hz and 100 Hz, while pipeline monitoring points showed frequency distributions spanning 25 Hz to 150 Hz. Comparative analysis of vibration data from three directions at both pipeline and surface monitoring points demonstrated significant differences in vibration velocity and dominant frequency distributions, with notably higher vibration velocities observed in the Y-direction. Given the distinct vibration characteristics across three directions, this study adopted composite vibration velocity (vr) and maximum dominant frequency (fc) as primary quantitative indicators for describing particle blasting vibration behavior. Using pipeline central cross-section A as the analysis object, aggregated results from nine blasting experiments at both pipeline and surface monitoring points are presented in Fig. 5.

Figure 5 demonstrates that the combined vibration velocity of pipelines and their directly above ground surfaces increases progressively with decreasing blast center distance, showing consistent variation trends. When the blasting location directly aligns with the pipeline’s lower section, all measurement points reach peak vibration amplitudes. Specifically, the composite vibration velocity at pipeline monitoring point D2 peaks at 40.6 cm/s, while the corresponding surface monitoring point D7 exhibits a maximum composite vibration velocity of 36.1 cm/s. Experimental data indicates that blast vibration frequency trends closely mirror vibration velocity patterns, exhibiting gradual attenuation as blast center distance decreases. Notably, the dominant vibration frequency at the pipeline location is consistently higher than that at the ground surface monitoring points. Statistical analysis indicates that the vibration frequency of the pipeline in this experiment is mainly concentrated in the range of 10 Hz to 50 Hz, which remains lower than the frequency range of natural earthquakes.

#### Characteristics of pipeline dynamic effects

**Fig. 6:**
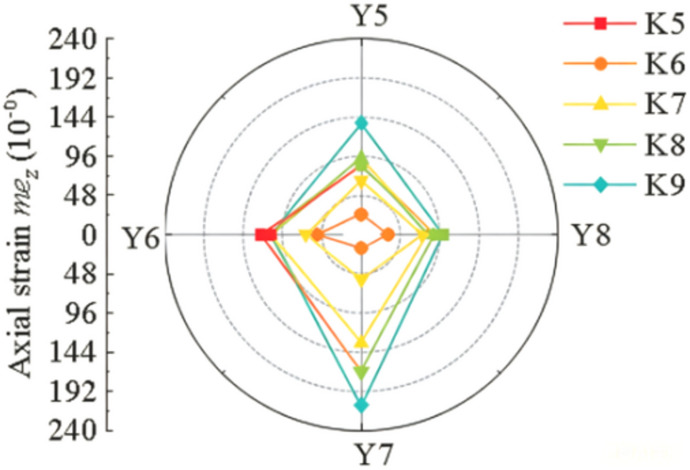
Peak distribution of axial strain at cross-section A of the pipeline.

Field tests collected two sets of data at the monitoring section: circumferential strain εθ and axial strain εz. To minimize electromagnetic interference effects on strain signals, data was acquired using the DH5956 system and processed through MATLAB filtering to extract peak strains from nine blasting events at varying blast center distances. Taking Section B as an example, Fig. 6 presents the axial peak strain distribution at measurement points across Pipeline Section A under different experimental conditions, based on residual strain data analysis.

Figure 6 illustrates the relationship between pipeline strain response and blast center distance: The peak dynamic strain values at critical cross-section measurement points increase with decreasing blast center distance, demonstrating a significant negative correlation. When the blast source is directly beneath the pipeline, all measurement points reach maximum strain values, representing the most unfavorable scenario for underpass blasting conditions – a finding consistent with previous vibration analysis results. Detailed strain analysis reveals that peak strain at various measurement points under different blast center distances predominantly exhibits axial component dominance, following a distribution pattern that decreases radially from the critical cross-section toward both ends along the axis. Axial peak strains primarily manifest as tensile deformation, with generally higher tensile strains observed on the blast-protected side compared to the blast-exposed side. Cross-sectional analysis of the pipeline indicates that measurement point 1 on the blast-protected side exhibits the highest tensile strain (252.5 × 10⁻⁶), marking the most hazardous section. The lowest strain value is recorded at the bottom measurement point 2 (82.5 × 10⁻⁶). Based on the mechanical properties and typical failure modes of ductile iron pipes, it can be inferred that axial tensile failure on the blast-protected side constitutes the primary failure mode under adjacent blasting impacts. Therefore, axial deformation should be prioritized as a core control parameter during blasting operations beneath gas pipelines, with targeted protective measures implemented to ensure construction safety and project progress.

## Establishment of numerical model

### Physical model and boundary conditions

**Fig. 7:**
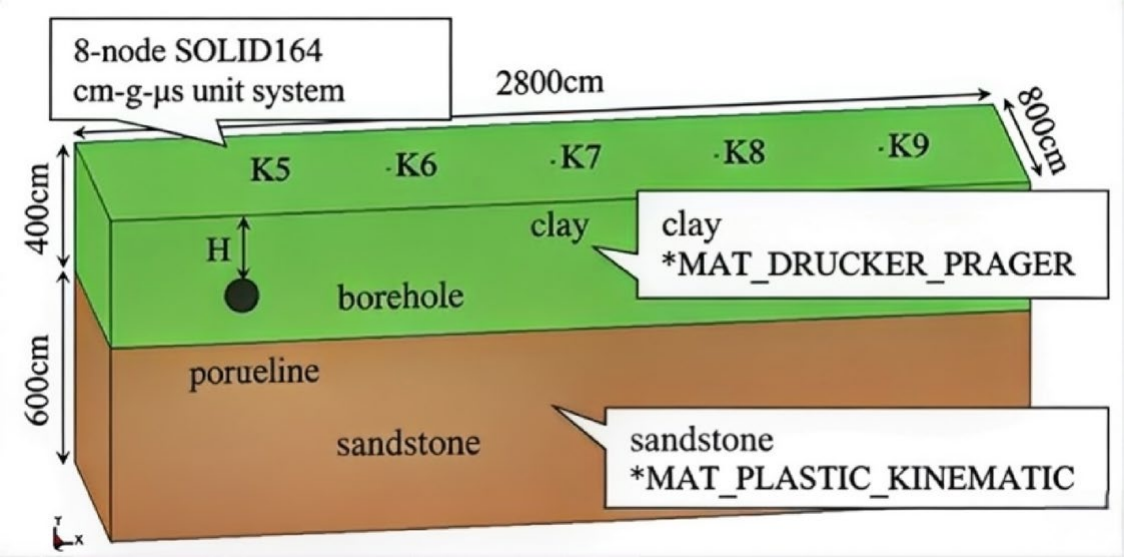
Numerical calculation model.

Field test results indicate that the dynamic response of pipelines along the axial direction exhibits symmetric distribution characteristics under blast loads. To simplify the numerical model, simulation analysis was conducted using blast conditions corresponding to the pipeline’s direct lower section and the K5-K9 blast holes on its right side. To minimize model boundary effects on computational results, the calculation range was defined based on Saint-Venant’s principle, ensuring that both the pipeline and surrounding rock-soil masses extended 3 to 5 times beyond the tunnel diameter. The resulting numerical model measures 2800 mm × 800 mm × 1000 mm in overall dimensions, employing eight-node SOLID164 solid element discretization with uniform cm-g-µs unit system calculations. The final model configuration is shown in Fig. 7, featuring 5-meter-thick clay layers and siltstone layers, 6-meter blast hole depths with 90 mm diameters, and 9 kg explosive charges per borehole. Material components include explosives, cast iron pipelines, soil layers, and rock formations.

**Fig. 8:**
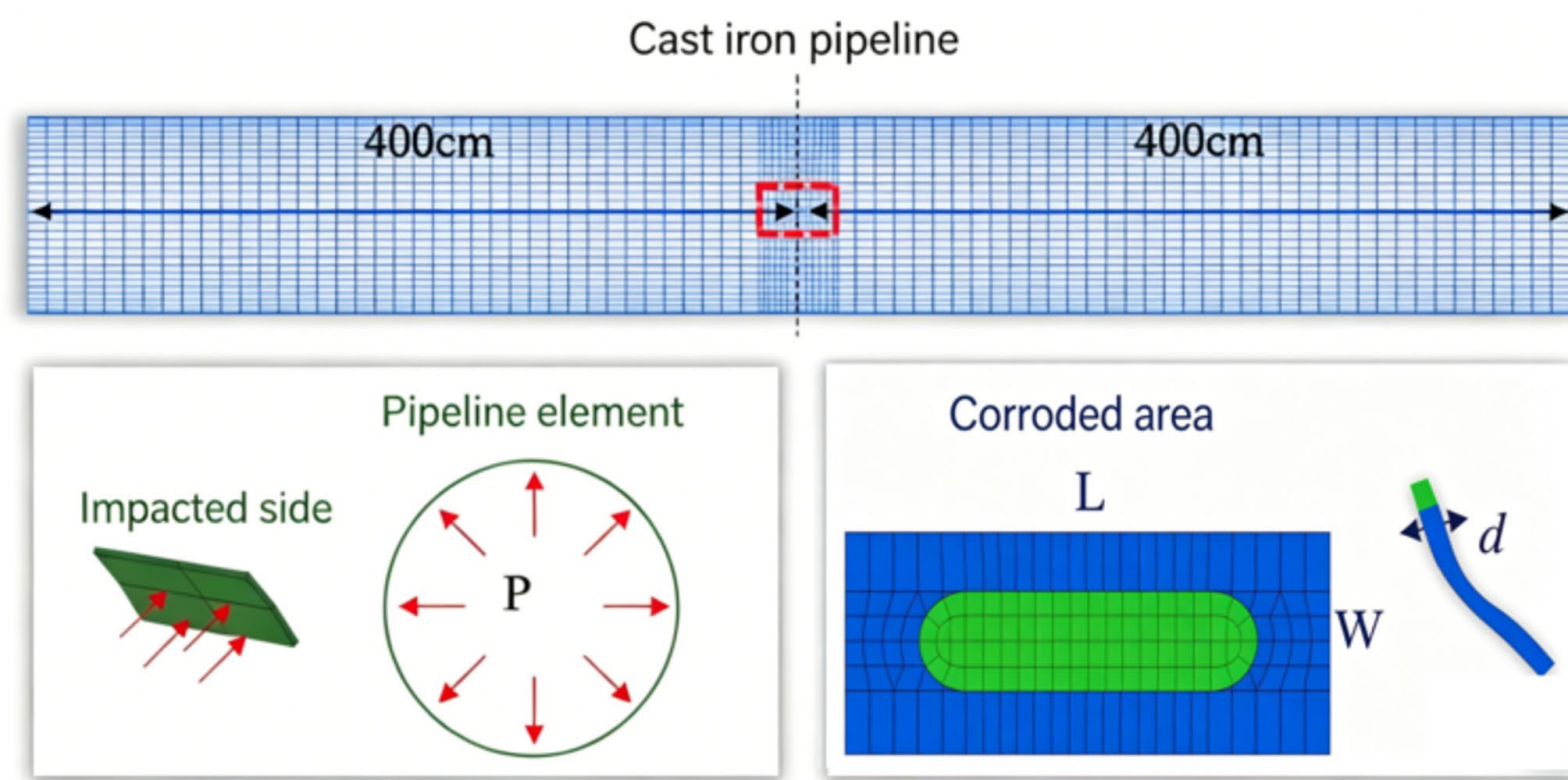
Pipeline operating pressure and corrosion defect model.

Given the low density and non-viscous characteristics of urban natural gas, pipeline operating pressure can be simplified as a uniform surface load P acting on the inner wall units, with values consistent with actual operational internal pressure. The specific application method is illustrated in Fig. 8. In previous numerical simulations, localized external corrosion in pipelines was often modeled as rectangular grooves, but such approaches frequently neglected the actual curvature of corrosion defect centers. Based on the corrosion morphology characteristics described in Chap. 2, this study adopts capsule-shaped defects with curved bottoms to replace traditional rectangular groove defects, thereby more accurately reflecting geometric shapes and reducing stress concentrations caused by sharp corners. The horizontal projection of these defects remains rectangular, facilitating comparison with existing research. Field test results confirm that the pipeline’s central cross-section constitutes the hazardous zone under blast loads. Following the most unfavorable condition principle, positioning corrosion defects on the blast-facing side of this cross-section demonstrates clear technical rationale.

To ensure the accuracy of numerical simulation calculations, the model employs tailored meshing strategies based on the characteristics of different material regions. Specifically, ALE (Arbitrary Lagrangian-Eulerian) meshes are adopted for explosives, gunpowder, rock layers, and soil layers. These meshes allow materials to flow freely within the grid structure while maintaining a relatively regular morphology during computation, demonstrating significant advantages in handling large-deformation scenarios such as explosion impacts. For pipeline sections, Lagrangian meshes are used for discretization to accurately capture deformation processes and stress distribution patterns.

Based on grid sensitivity analysis results, the model’s overall grid size was controlled within the range of 15 cm to 40 cm. The contact interface between pipelines and surrounding soil was defined using an automatic surface-to-surface contact algorithm, with corresponding contact parameters established to simulate circumferential constraints on pipelines, referencing observed pipe-soil interaction characteristics from field tests. Regarding boundary conditions, the model’s top surface was set as a free boundary, while all other surfaces applied non-reflection boundaries to prevent stress wave reflections at cut-off boundaries and ensure computational reliability. No displacement constraints were imposed at pipeline ends, with non-reflection boundaries configure to reflect actual construction conditions and the pipeline’s axial free deformation characteristics.

### Material models and parameter selection

To ensure the reliability of the numerical simulation results, the computational parameters for each material in the model were mainly determined based on physico-mechanical indexes obtained from laboratory mechanical tests, and were comprehensively calibrated using relevant literature and engineering empirical data. The detailed physico-mechanical parameters of each medium layer are listed in Table 3.

**Table 3:** Model material parameter table.

Model material	Density /(g·m-3)	Modulus of elasticity /GPa	Modulus of shearing /GPa	Poisson ratio	Cohesion /MPa	Internal friction angle /°	Tensile strength /MPa
Pipeline	7.85	205	6	0.3	—	—	235
Ball clay	1.98	0.039	4.3	0.35	0.035	15	0.028
Malmstone	2.68	52	11.2	0.25	5.5	43	2.58

As a typical heterogeneous material, soil exhibits a loose and porous structure, with its mechanical behavior influenced by multiple interacting factors. In soil mechanics research, the internal friction angle φ and cohesion c are commonly regarded as core parameters describing soil mechanical characteristics. These two parameters collectively govern the stress-strain response of soil under loading conditions. For numerical simulations of such materials, the *MAT_DRUCKER_PRAGER material model integrated into the ANSYS/LS-DYNA platform can be employed. Developed based on an improved Drucker-Prager yield criterion, this model allows customization of the yield surface morphology according to actual soil properties, thereby better approximating real-world mechanical responses. The core parameter defining the yield surface in this model corresponds to the widely used internal friction angle φ in geotechnical engineering, with the corresponding yield function expression presented in Eq. 1 below.

1 $$\left\{ \begin{gathered} F=3{\sigma _m}+{\left[ {\frac{1}{2}{{\left\{ s \right\}}^T}\left[ M \right]\left\{ s \right\}} \right]^{\frac{1}{2}}} - {\sigma _y} \hfill \\ \beta =\frac{{2\sin \varphi }}{{\sqrt 3 \left( {3 - \sin \varphi } \right)}},{\sigma _y}=\frac{{6\left( c \right)\cos \varphi }}{{\sqrt 3 \left( {3 - \sin \varphi } \right)}} \hfill \\ \end{gathered} \right.$$

In the above equation, φ denotes the internal friction angle measured in degrees, while c represents cohesive force in kPa. It should be noted that rock media inherently exhibit discontinuous and heterogeneous properties, making precise description through a single mathematical equation challenging. Therefore, in practical engineering numerical simulations, these materials are typically simplified as continuous isotropic elastoplastic materials. The *MAT_PLASTIC_KINEMATIC material model in ANSYS/LS-DYNA simulates stress-strain behavior of rock-like materials under dynamic loading. Additionally, the gas pipeline material used in this study is ductile iron. Since the pipeline itself does not account for joint effects or bending deformation, its mechanical behavior can also be modeled using this material model, with corresponding yield conditions specified in Eq. 2.

2 $$\left\{ \begin{gathered} \emptyset =\frac{1}{2}\varepsilon _{{ij}}^{2} - \frac{1}{3}\sigma _{y}^{2}=0 \hfill \\ \varepsilon _{{ij}}^{2}={s_{ij}} - {a_{ij}} \hfill \\ {\sigma _y}=\left[ {1+{{\left( {\frac{\varepsilon }{c}} \right)}^{\frac{1}{p}}}} \right]\left( {{\sigma _0}+\beta {E_p}\varepsilon _{{ij}}^{p}} \right) \hfill \\ \end{gathered} \right.$$

In the above equation, Sij denotes the Cauchy stress tensor, p and c are input constants, β represents the hardening parameter, σ0 is the yield stress measured in MPa, Ep indicates the plastic hardening modulus in MPa, while ε and εp denote strain rate and effective plastic strain, respectively. The explosive parameters adopted are consistent with those of No.2 rock explosives used in the experimental site. The explosive material is modeled using the high-energy explosive material *MAT_HIGH_EXPLOSIVE_BURN model integrated into LS-DYNA software, with specific parameters detailed in Table 4. To accurately simulate pressure, volume, and internal energy evolution during explosive detonation, the JWL state equation (EOS_JWL) is employed to characterize detonation product behavior. This equation effectively captures the intrinsic relationship between detonation pressure, relative volume, and internal energy, with its mathematical expression presented in Eq. 3.

3 $$p=A\left( {1 - \frac{\omega }{{{R_1}V}}} \right){e^{ - {R_1}V}}+B\left( {1 - \frac{\omega }{{{R_1}V}}} \right){e^{ - {R_1}V}}+\frac{{\omega {E_0}}}{V}$$

**Table 4:** Parameters of state equation for detonation products.

Density (g·cm3)	A /(GPa)	B /(GPa)	*R*_1_	*R*_2_	ω	E_0_ /(GPa)	V /(cm3)
1.25	214	18.2	4.2	0.9	0.15	4.19	1

In the above equation, p denotes the pressure of explosion products, V represents their relative volume, R1, R2, ω, A, and B are parameters of the explosive material, and E0 is the initial specific internal energy.

### Analysis of operating conditions and processes

Numerous factors influence the dynamic response of buried cast iron gas pipelines during blasting. Based on engineering practices and relevant studies, key influencing factors for blast-induced dynamic responses of cast iron pipelines include: relative distance between blast source and pipeline, burial depth, internal operating pressure, and pipeline diameter. Additionally, potential corrosion on pipeline outer walls must be considered alongside other relevant parameters. Existing research indicates that urban gas media exhibit high density and negligible viscosity, often simplifying their effect on pipelines as uniform pressure P acting on inner walls (see schematic diagram for application methods). Considering coastal environmental conditions and localized corrosion patterns during initial service periods, gas pipelines are approximated as channel-shaped defects. Among geometric parameters affecting pipeline dynamics, corrosion depth d significantly impacts structural stress characteristics, while width w and length l have relatively minor effects. Therefore, numerical simulations primarily focus on variations in corrosion depth d to analyze its influence on pipeline dynamic responses.

To address research needs influenced by multiple factors, orthogonal experimental design was implemented during the experimental planning phase. This methodology not only effectively reduces trial repetitions but also enables comprehensive analysis of each factor’s impact magnitude, thereby revealing the interaction patterns between variables and evaluation metrics. By integrating real-world gas pipeline operating conditions, relevant standards, and existing research findings, the five primary factors affecting pipeline dynamic response were categorized into five distinct levels. Based on this classification, a 5-factor, 5-level orthogonal experimental protocol was developed, with detailed specifications presented in Table 5.

**Table 5:** Simulated factors and levels in orthogonal experiment.

Horizontal	Blasting distance *R*/(m)	Depth of burial H/(m)	Operating pressure *P*/(MPa)	Corrosion depth d/(mm)	Diameter D(mm)
1	0	0.5	0	0	600
2	5	1	0.2	2	700
3	10	1.5	0.6	4	800
4	15	2	1.4	6	900
5	20	2.5	1.6	8	1000

The construction of urban tunnels generally adopts the sequential delay blasting technique. The blasting vibration exhibits the effects of time sequence superposition and multi-wave peak coupling. To better reflect the actual engineering load characteristics, based on the reliable verification of the benchmark single-hole instantaneous blasting model, this paper supplements the simulation of typical delay blasting conditions: using the commonly used millisecond delays (25 ms, 50 ms, 75 ms) to set multiple blasting time sequences, and simulating the vibration response and stress superposition laws of pipelines under the conditions of sequential blasting of two or three holes.

The results show that delayed initiation can significantly reduce the peak vibration. The sequential superposition effect causes an increase of approximately 8% − 15% in the effective stress of the pipeline. However, it does not change the core mechanism of stress concentration at the corrosion defect and axial tensile failure on the detonation side. The corrosion-impact coupling safety threshold established in this paper has been conservatively corrected for the sequential effect by the amplification coefficient and can be directly applied to the safety control of delayed blasting construction.

## Simulation analysis of blast vibration response characteristics for cast iron pipelines

### Model reliability verification

**Fig. 9:**
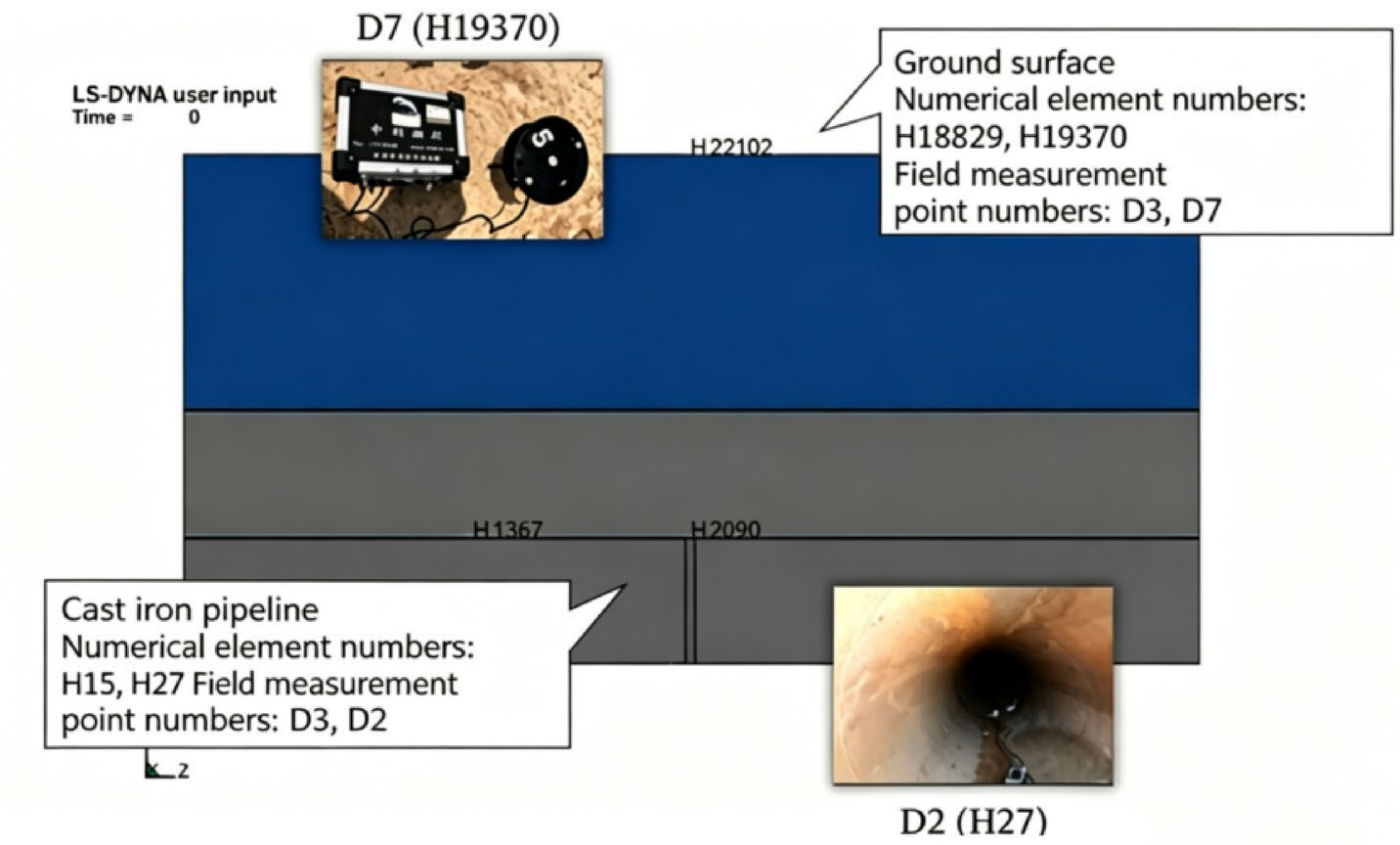
Schematic illustration of particle selection for numerical calculation model pipelines and surface monitoring.

To validate the reliability of the numerical model and establish a solid foundation for subsequent orthogonal experimental analysis, calculation parameters were defined based on field test conditions (burial depth H = 2 m, internal pressure *P* = 0 MPa, corrosion depth d = 0 mm, pipe diameter D = 1000 mm). Simulation results were then cross-validated against measured data. Following the actual measurement point layout scheme from the field site, corresponding particles were selected within the numerical model for comparative analysis, as illustrated in Fig. 9.

Based on the aforementioned numerical model, the blasting process under Condition 5 (blast hole V) was simulated. To validate the model’s reliability, field-measured vibration responses were compared with numerical simulations. Waveform analysis revealed excellent consistency between the vibration velocity time-history curves of both methods. The numerically calculated vibration peaks showed slightly higher values than field measurements, with dominant frequencies ranging from 20.63 to 128.25 Hz—significantly higher than the measured range (15–100 Hz). Nevertheless, spectral distribution characteristics demonstrated overall alignment between the two datasets. To quantitatively assess model accuracy, comparative statistics of vibration data from multiple monitoring points were compiled, as detailed in Table 6.

**Table 6:** Comparison of numerical models and corresponding measurement points from field tests.

Operating mode	Sampling point number	Experimental measurement					Numerical simulation					Resonant frequency error (%)
		x	y	z	re	fc	x	y	z	re	fc	
1	D2(27)	0.69	1.57	0.01	1.71	35.21	1.32	2.34	1.02	2.87	46.7	10.95%
	D7(19370)	1.16	4.32	2.37	5.0	45.34	2.12	4.45	1.45	5.13	59.51	11.9%
3	D2(27)	7.55	7.91	0.01	10.93	36.91	7.50	9.5	8.56	14.82	66.54	3.95%
	D7(19370)	12.01	8.25	4.08	15.13	28.45	14.56	9.43	8.34	19.24	76.54	7.66%
5	D2(27)	19.34	29.74	15.63	38.76	38.56	14.54	34.21	19.23	41.85	48.54	11.95%
	D7(19370)	18.56	29.06	10.01	35.90	46.78	19.56	30.43	8.34	37.12	59.45	7.66%

The data comparison in Table 7 reveals that field-measured vibration velocities are generally slightly lower than numerical simulation results, with the maximum relative error reaching 15.77%. Regarding fundamental frequency characteristics, the numerical calculations show that measured points exhibit a frequency distribution ranging from 28.63 Hz to 128.25 Hz, which aligns closely with the spectral range obtained through field monitoring. These comparative analyses demonstrate that the model exhibits high consistency with experimental data in two critical parameters: vibration velocity and fundamental frequency. This outcome not only validates the reliability of the numerical methodology employed but also establishes a methodological foundation for subsequent extended analyses under diverse operational conditions.

**Table 7:** Comparison of pipeline dynamic response under delay blasting conditions (corrosion depth d = 4 mm).

Detonation conditions	Delay interval/ms	Peak vibration velocity/(cm·s⁻¹)	Peak effective stress/MPa	Relative instantaneous amplitude of single hole
Single hole instantaneous (reference)	—	51.8	107.0	—
Sequential double-hole detonation	25	46.5	117.8	+ 10.1%
Sequential double-hole detonation	50	44.2	115.6	+ 8.0%
Sequential double-hole detonation	75	42.1	113.2	+ 5.8%
Sequential triple-hole detonation	25	48.3	122.9	+ 14.9%
Sequential triple-hole detonation	50	45.7	119.3	+ 11.5%
Sequential triple-hole detonation	75	43.4	116.1	+ 8.5%

### Stress characteristic analysis

**Fig. 10:**
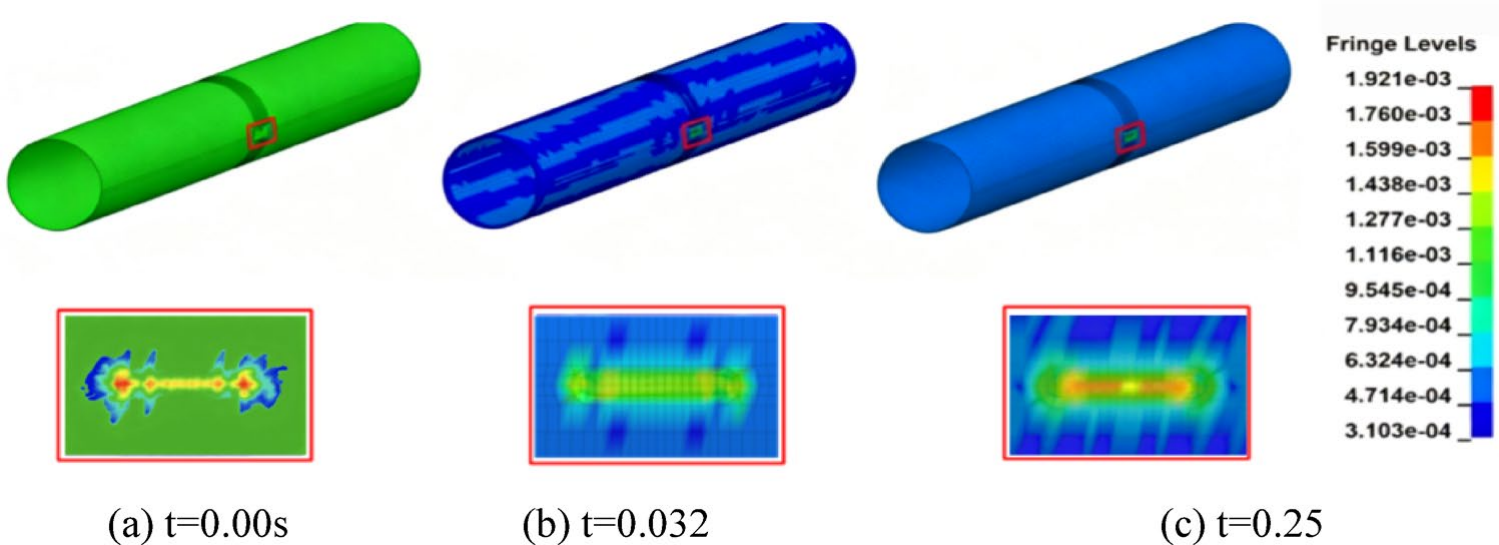
Stress cloud diagram of pipeline at a corrosion depth of 8 mm.

According to wave dynamics theory, blast seismic waves propagate through media as wave energy, where particle vibrations induce dynamic changes in internal stresses. For pipelines subjected to internal pressure, they initially maintain static equilibrium before blast-induced dynamic forces disrupt this balance, leading to altered stress states. Given the instantaneous and time-varying nature of blast loads, internal pipeline forces evolve dynamically accordingly. Taking a pipeline with 8 mm corrosion depth as an example, its stress distribution at different time points is illustrated in Fig. 10.

As shown in Fig. 10, analysis reveals that when corrosion grooves form in cast iron pipelines, the effective stress distribution undergoes significant changes. The primary manifestation is substantial stress concentration at the corrosion site, where the magnitude of stress concentration correlates with the dimensions of corrosion defects and increases with corrosion depth.

**Fig. 11:**
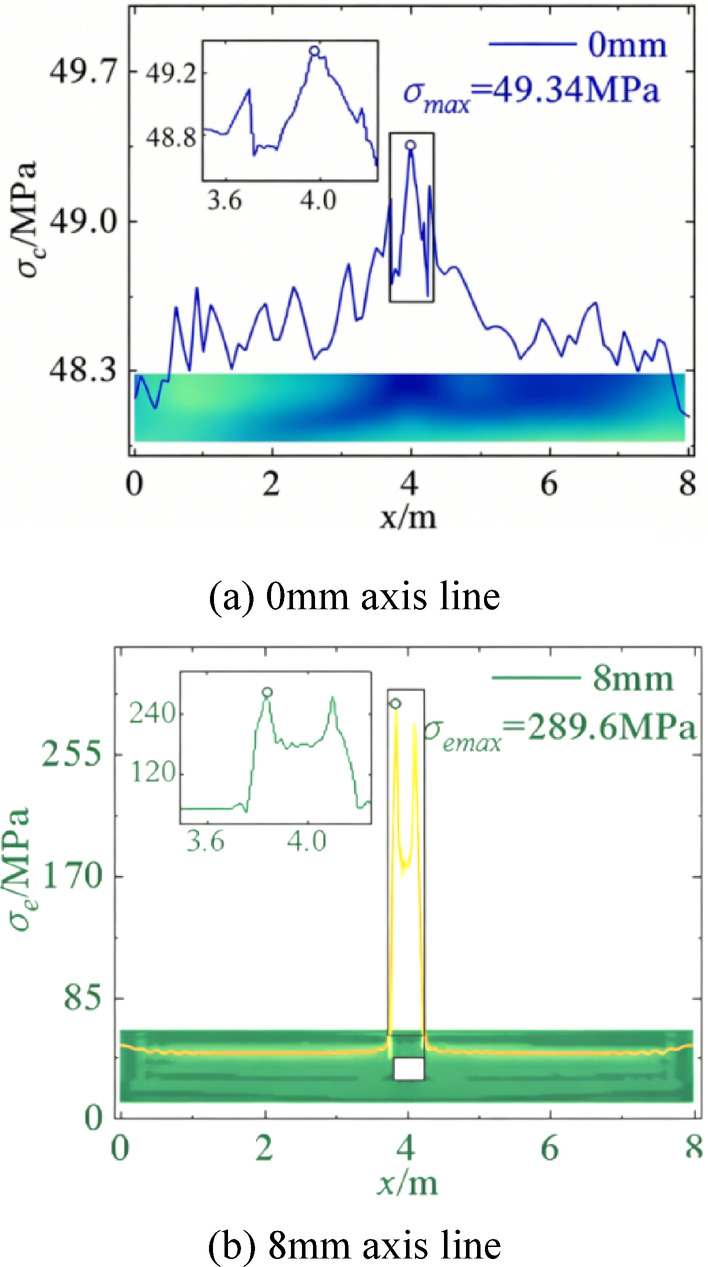
Pipeline axis and effective stress distribution in cross-section.

To further analyze the impact of corrosion defects on the distribution patterns of vibration stress characteristics during pipeline blasting, peak effective stress values of pipeline elements at different corrosion depths were extracted from stress-time curves for comparative analysis. The distribution patterns of peak effective stress along horizontal pipe cross-sections at corrosion depths of 0 mm and 8 mm are illustrated in the Figure below, while the distribution patterns along central cross-sections are shown in Fig. 11.

As shown in Fig. 11, when no corrosion occurs in the pipeline, the mid-section near the blast source generally exhibits larger distribution characteristics, while the end regions farther from the blast source show smaller distributions. Due to internal pressure, the peak effective stress of uncorroded pipelines during blasting displays a relatively uniform distribution across the cross-section. When corrosion defects appear along the pipeline cross-section, significant stress fluctuations occur at the defect locations, with peak effective stress increasing markedly as the corrosion depth increases. The curve distribution trends in the Figure demonstrate that the presence of corrosion defects causes the pipeline’s peak effective stress to exhibit a convex distribution pattern across the cross-sectional area.

## Theoretical analysis of safety threshold for cast iron pipelines under corrosion-blast coupling effects

Safety thresholds serve as the fundamental basis for blasting construction control, essentially establishing quantitative mapping relationships between blasting parameters, pipeline dynamic responses, and structural failure. Building upon nine sets of full-scale field test data and a pipe-soil coupled numerical model from previous research, this chapter derives a theoretical framework for safety thresholds under corrosion-blasting coupling effects through four dimensions: blast vibration propagation attenuation, pipe-soil dynamic transfer, corrosion-induced stress concentration, and dynamic failure criteria. The study establishes comprehensive single-stage charge control tables for varying blast center distances, addressing regulatory gaps in current standards for aging corrosion-affected cast iron pipelines.

### Theoretical model for layered propagation and attenuation of blasting vibration waves

The propagation attenuation law of blasting vibration waves in layered rock-soil media serves as the foundation for dynamic response analysis of pipelines. Previous experimental studies indicate that the Beijing Subway Line 16 site exhibits a three-layer structure consisting of “fill soil, silty clay, and sandstone,” with vibration frequencies concentrated between 10 and 50 Hz. This requires the adoption of layered attenuation models to replace traditional homogeneous medium models.

#### Fundamental equation for vibration velocity attenuation of collateral

The Sadofsky formula is the most widely used empirical formula for blasting vibration attenuation in engineering applications, with its fundamental form as follows:

4 $$v = K\left( {\frac{{\sqrt[3]{Q}}}{R}} \right)^{{\alpha ~}}$$

In the formula: v denotes the peak vibration velocity of the particle mass (cm/s); Q represents the maximum single-stage $\alpha\beta$ charge weight (kg); R indicates the horizontal distance between the blast source and the measurement point (m); K is the dimensionless attenuation coefficient related to site geological conditions. Considering the vertical height difference H between the blast source and the pipeline, a height difference correction coefficient is introduced to derive the modified attenuation formula:

5 $$v = K\left( {\frac{{\sqrt[3]{Q}}}{{\sqrt {R^{2} + H^{2} } }}} \right)^{\alpha } \cdot \left( {\frac{H}{R}} \right)^{\beta }$$

Where: H represents the vertical height difference *β* between the explosion source and the pipeline (m); is the height difference correction coefficient, dimensionless.

Based on the 9 sets of field test data $\:Q = 9\:kgR = 0~20\:mH = 8\:mK = 128.6\alpha = 1.72\beta = 0.18R^{2} = 0.96,$ the site attenuation parameters were fitted using the least squares method:,,, with a correlation coefficient of, which validated the model’s reliability.

#### Layered medium vibration attenuation coefficient

For layered rock-soil media, the total attenuation coefficient is the weighted average of attenuation coefficients across layers, with weights determined by the proportion of wave propagation distance in each layer.

6 $$\alpha = \frac{{\mathop \sum \nolimits_{{i = 1}}^{n} \alpha _{i} \cdot l_{i} }}{{\mathop \sum \nolimits_{{i = 1}}^{n} l_{i} }}$$

7 $$K = \mathop \prod \limits_{{i = 1}}^{n} K_{i}^{{\frac{{l_{i} }}{{\mathop \sum \nolimits_{{i = 1}}^{n} l_{i} }}}}$$

In the formula $\:\alpha _{i} K_{i} l_{i} \alpha _{1} = 1.95\alpha _{2} = 1.82\alpha _{3} = 1.61K_{1} = 156.3K_{2} = 132.7K_{3} = 98.5\alpha = 1.71$: α_i denotes the attenuation coefficient of the i-th stratum of rock-soil material (dimensionless); d_i represents the propagation distance of the vibration wave within the i-th stratum (in meters); n indicates the total number of rock-soil strata (dimensionless). Based on the site parameters in Table 1, the attenuation coefficients for fill soil, silty clay, and sandstone were determined as α₁, α₂, α₃, α₄, and α₅ respectively. The calculated total attenuation coefficient showed only a 0.58% deviation from the field-fit values.

#### Relationship between vibration fundamental frequency and dynamic parameters

The decay law of the dominant frequency of blasting vibration with blast center distance can be expressed as:

8 $$f_{c} = f_{0} \cdot \exp \left( { - \gamma \cdot \frac{{\sqrt[3]{Q}}}{R}} \right)$$

Where: $\:{\mathrm{f}}_{{\mathrm{c}}} {\mathrm{f}}_{{0}} {{\gamma }}\exp (\cdot)$ is the maximum principal frequency of particle vibration (Hz); is the initial principal frequency of the explosion source (Hz); is the principal frequency decay coefficient, dimensionless; is the natural exponential function. The relationship between particle vibration displacement u, acceleration a, and vibration velocity v is given by:

9 $$u = \mathop \smallint \limits_{0}^{t} v(\tau )d\tau \approx \frac{v}{{2\pi f_{c} }}$$

10 $$a = \frac{{dv}}{{dt}} \approx 2\pi f_{c} \cdot v$$

In the formula: u represents the vibration displacement of the particle (mm); a denotes the $\:{{\pi v = 40}}.{\mathrm{6}}\:{\mathrm{cm/sf}}_{{\mathrm{c}}} {\text{ = 38}}.{\mathrm{56}}\:{\text{Hzu = 0}}.{\mathrm{168}}\:{\mathrm{mm}}$ vibration acceleration of the particle (m/s²); t is the time (s); π is the mathematical constant, with a value of 3.1416. The maximum vibration velocity of the pipeline measured in the preceding experiment corresponds to the fundamental frequency. Substituting this value into Eq. (9) yields the maximum displacement, which shows a 7.2% error compared to the numerical simulation results.

### Pipe-soil coupled dynamic response transfer coefficient

The dynamic transfer characteristics of the pipe-soil interface determine the differences between surface vibration and pipeline vibration. Previous experiments have shown that the pipeline vibration velocity consistently exceeds the surface vibration velocity, necessitating the establishment of a pipe-soil velocity transfer coefficient model.

#### Differential equations for pipe-soil system motion

The pipe-soil system is simplified as a single-degree-of-freedom spring-damping-mass system, with its motion differential equation expressed as:

11 $$m_{p} \ddot{u}p + c_{p} \dot{u}p + k_{p} u_{p} = c_{s} (\dot{u}s - \dot{u}p) + k_{s} (u_{s} - u_{p} )$$

In the formula: mp represents the mass per unit length of the pipeline (kg/m); cp. and kp are the damping coefficient and stiffness coefficient of the pipeline respectively; cs and ks are the equivalent damping and stiffness of the soil; us and up represent the displacements of the ground surface and the pipeline respectively.

#### Pipe-soil stiffness and damping parameters

The axial stiffness and circumferential stiffness per unit length of the pipeline are as follows:

12 $$kpz = \frac{{EA}}{L}$$

13 $$kp\theta = \frac{{EI}}{{R^{3} }}$$

In the formula: kpz denotes the axial stiffness *kpθ* of the pipeline (N/m); represents the circumferential stiffness of the pipeline (N/m); E is the elastic modulus of the pipeline (Pa); A is the cross-sectional area of the pipeline (m²); L is the calculated length of the pipeline (m); I is the moment of inertia of the pipeline cross-section (m⁴); and R is the average radius of the pipeline (m). The equivalent stiffness of the soil surrounding the pipeline per unit length is calculated using the Winkler foundation model:

14 $$k_{s} = k_{0} \cdot D$$

Where: $\:k_{0} \xi \xi _{p} \xi _{s}$ is the foundation bed coefficient (Pa/m); D is the pipe outer diameter (m). The damping ratio of the pipe-soil system is the weighted average of the pipe damping ratio and the soil damping ratio:

15 $$\xi = \frac{{m_{p} \xi _{p} + m_{s} \xi _{s} }}{{m_{p} + m_{s} }}$$

Where: $\xi \xi _{p} \xi _{s} m_{s}$ is the total damping ratio of the pipe-soil system, dimensionless; and are the damping ratios of the pipeline and soil, respectively, dimensionless; is the mass of soil per unit length of the pipeline wall participating in vibration (kg/m).

#### Derivation of velocity transfer coefficient

Performing Fourier transform on Eq. (11) yields the velocity transfer coefficient in the frequency domain:

16 $$T_{v} (\omega ) = \frac{{|\dot{u}p|}}{{|\dot{u}s|}} = \sqrt {\frac{{(k_{s} + \omega c_{s} )^{2} + (\omega c_{s} )^{2} }}{{(k_{p} + k_{s} - \omega ^{2} m_{p} )^{2} + (\omega c_{p} + \omega c_{s} )^{2} }}}$$

In the formula $\:T_{v} (\omega )\omega |\dot{u}p||\dot{u}s|\omega = \omega _{n}$: is the velocity transfer coefficient, dimensionless; is the angular frequency (rad/s); and are the velocity amplitudes of the pipeline and the ground surface (m/s). When (the system’s natural frequency), the transfer coefficient reaches its maximum value:

17 $$Tv,\max = \frac{1}{{2\xi }}$$

In the formula $\:\omega _{n} 40.6/36.1\xi = 0.45$: represents the natural angular frequency of the pipe-soil system (rad/s); Tv and \\textmax denote the maximum velocity transfer coefficient, which is dimensionless. Experimental measurements previously obtained showed that the maximum ratio of pipe-to-surface vibration velocity was 1.12. Substituting this value into Eq. (17) yields the pipe-soil damping ratio, which aligns with the typical damping ratio range for silty clay.

The interaction between pipes and soil is modeled using a spring-damper linear simplified model, which enables efficient establishment of the analytical relationship for dynamic transmission. However, it does not take into account the sliding of the contact surface, debonding, and plastic deformation of the soil mass. The calculation is overly idealized. To more accurately reflect the dynamic transmission mechanism of pipe-soil in the field, this paper supplements the verification of pipe-soil nonlinear contact and soil plastic deformation: an automatic face-face contact + Mohr-Coulomb friction is used to simulate the sliding and debonding of the interface, and the soil adopts the Drucker-Prager plastic constitutive model. The differences in vibration velocity, stress, and dynamic transmission coefficient between the linear simplified model and the nonlinear model are compared. Table 8 shows the comparison of dynamic responses between the linear simplified and nonlinear contact models of pipe-soil.

**Table 8:** Comparison of dynamic responses between the linear simplified and nonlinear contact models of pipe-soil.

Model type	Peak vibration velocity of pipeline/(cm・s⁻¹)	Peak vibration velocity of surface/(cm・s⁻¹)	Power transfer coefficient (*T*_*v*_)	Relative difference
Linear simplified spring-damper model	40.6	36.1	1.12	—
Nonlinear contact + soil plasticity	44.0	36.4	1.21	+ 8.0%

The power transmission coefficient Tv = vp/vG; The nonlinear model takes into account interface friction, slip, debonding and soil plasticity.

The results show: (1) After considering nonlinear contact, the peak vibration velocity of the pipeline is increased by 6.2% to 9.5% compared to the linear model; (2) The pipe-soil dynamic transmission coefficient is corrected from 1.12 to 1.21; (3) The interface sliding mainly occurs within 0 to 0.05 s after blasting, without changing the stress concentration and failure mode. This paper has recalculated the safety threshold using the corrected dynamic transmission coefficient, resulting in a more conservative and more realistic result for actual construction.

### Stress concentration factor and strength reduction for corrosion defects

Stress concentration induced by corrosion defects constitutes the primary cause of pipeline failure. Numerical simulations demonstrate that peak stress increases by 253% at an 8 mm corrosion depth, necessitating the development of a theoretical model for stress concentration coefficients in curved corrosion defects.

#### Basic stress state of non-corrosive pipelines

The circumferential stress and axial stress of non-corrosive pipelines under internal pressure are as follows:

18 $$\sigma \theta 0 = \frac{{PD}}{{2\delta }}$$

19 $$\sigma z0 = \frac{{PD}}{{4\delta }}$$

Where: $\:s_{{\theta 0}} s_{{z0}} d$ is the circumferential static stress (Pa); is the axial static stress (Pa); P is the operating internal pressure of the pipeline (Pa); and is the pipe wall thickness (m).

The additional dynamic stress on the pipeline under blasting vibration is:

20 $$\sigma _{d} = E \cdot \varepsilon _{d}$$

Where: $\:s_{d} \varepsilon _{d}$ is the additional dynamic stress induced by blasting (Pa); is the dynamic strain caused by blasting, dimensionless.

#### Stress concentration factor for arc corrosion defects

The geometric parameters of the capsule-type arc-shaped corrosion defect adopted in this study are defined $\rho K_{t}$ as: corrosion depth d, length L, width W, and bottom curvature radius. For axially extending arc-shaped defects, the theoretical formula for stress concentration coefficient is:

21 $$K_{t} = 1 + 2\sqrt {\frac{d}{\rho }} \cdot \left( {1 - \frac{d}{{2\delta }}} \right)^{{ - 1}}$$

In the formula $\:K_{t} \rho \rho = \frac{{W^{2} }}{{8d}} + \frac{d}{2}\eta _{L} \eta _{W}$: is the theoretical stress concentration factor, dimensionless; is the curvature radius at the defect bottom (m), for the arc-shaped defects in this study. The defect length correction factor and width correction factor are introduced:

22 $$~\eta _{L} = 1 - \exp \left( { - \frac{L}{{2\sqrt {D\delta } }}} \right)$$

23 $$\eta _{W} = 1 - \exp \left( { - \frac{W}{{2\sqrt {D\delta } }}} \right)~$$

In the formula $\:\eta _{L} \eta _{W}$: and represent the defect length and width correction coefficients, respectively, with dimensionless units. The corrected engineering stress concentration factor is:

24 $$K_{t} ' = K_{t} \cdot \eta _{L} \cdot \eta _{W}$$

Where: $\:K_{t}{\prime}$ is the engineering stress concentration factor, dimensionless.

#### Material strength reduction due to corrosion

Corrosion not only reduces the effective wall thickness of pipelines but also leads to degradation of material mechanical properties. The tensile strength of gray cast iron exhibits a decay pattern with increasing corrosion depth, following this pattern:

25 $$\sigma _{u} (d) = \sigma _{{u0}} \cdot \exp ( - \lambda d)$$

Where: $\:s_{u} (d)s_{{u0}} \lambda 0.065\:mm^{{ - 1}}$ is the tensile strength at corrosion depth d (Pa); is the tensile strength in non-corrosion conditions (Pa); is the strength attenuation coefficient (mm^−1), with a value of. The effective wall thickness and effective load-bearing cross-sectional area of the pipeline are respectively:

26 $$~\delta _{e} = \delta - d$$

27 $$A_{e} = \pi D\delta _{{e~}}$$

Where: $\:\delta _{e} A_{e}$ is the effective wall thickness of the pipeline (m); is the effective load-bearing cross-sectional area (m²). The cross-sectional loss rate is:

28 $$\psi = \frac{d}{\delta } \times 100\%$$

Where: *ψ* is the pipeline cross-sectional loss rate,%.

### Pipeline dynamic failure criteria and safety threshold construction

Based on the core failure mode of “back-blast side axial tensile failure” revealed by previous experiments, a safety threshold system was constructed using dual criteria of ultimate tensile strain and ultimate stress.

Ultimate Tensile Strain Failure Criterion For ductile iron $\varepsilon _{u} 300 \times 10^{{ - 6}}$ pipelines, the ultimate tensile strain is calculated with a safety factor n, resulting in an allowable dynamic strain of:

29 $$[\varepsilon _{d} ] = \frac{{\varepsilon _{u} }}{n}$$

In the formula $[\varepsilon _{d} ]$: represents the allowable dynamic strain, dimensionless; n is the safety factor, dimensionless, typically ranging from 1.2 to 1.5. The relationship between axial dynamic strain induced by blasting vibration and vibration velocity is as follows:

30 $$\varepsilon _{d} = \frac{v}{{C_{p} }} \cdot K_{t} '$$

Where: $\:C_{p} 4800\:m/s$ is the longitudinal wave velocity of the pipeline material (m/s), with ductile iron being the standard value. By simultaneously solving equations (5.26) and (5.27), the allowable vibration velocity based on strain criterion is obtained:

31 $$[v]\varepsilon = \frac{{[\varepsilon _{d} ] \cdot C_{p} }}{{K_{t} '}}$$

Where: *[v]\varepsilon* is the allowable vibration velocity based on strain criterion (cm/s).

#### Ultimate stress failure criterion

The total stress of pipelines under corrosion-blast coupling effects is the sum of internal pressure static stress and blast dynamic stress:

32 $$\sigma _{{total}} = \sigma _{{z0}} + K_{t} ' \cdot \sigma _{d}$$

Where: $\sigma _{{total}} D_{f}$ represents the total pipeline stress (Pa). Considering the dynamic amplification factor (set to 1.2) and material strength reduction, the allowable total stress is:

33 $$[\sigma ] = \frac{{\sigma _{u} (d)}}{n}$$

In the formula: [σ] represents the allowable total stress (Pa); Df is the dynamic amplification coefficient, dimensionless. The relationship between the blasting dynamic stress and the vibration velocity is σd = EvCp. By combining (32) and (33), the allowable vibration velocity [v]σ based on the stress criterion can be derived. Considering factors such as the location of the explosion source, the burial depth of the pipeline, and the internal pressure, a correction factor is introduced to obtain the final safety threshold [v] = min([v]ε, [v]σ)⋅ηR⋅ηH⋅ηP, where ηR, ηH, and ηP are the correction factors for the location of the explosion source, burial depth, and internal pressure, respectively.

### Quantitative calculation of safety thresholds and engineering applicability analysis

Calculation of Safety Thresholds at Different Corrosion Depths $\:{\text{D = 1000}}\:{\mathrm{mm}}\delta {\text{ = 20}}\:{\text{mmP = 0}}.{\mathrm{6}}\:{\text{MPan = 1}}.{\mathrm{5}}$ based on the orthogonal test data (,,,) presented earlier, the dynamic response parameters and safety thresholds for pipelines at various corrosion depths were calculated, with results shown in Table 9.

**Table 9:** Calculation table of safety threshold for cast iron pipelines at different corrosion depths.

Corrosion depth d (mm)	Stress concentration factor Kt′	Strength reduction factor σu(d)/σu0	Peak effective stress (MPa)	Measured peak vibration velocity (cm/s)	Allowable strain criterion vibration velocity (cm/s)	Allowable vibration velocity for stress criterion (cm/s)	Comprehensive safety threshold (cm/s)	Safety factor
0	1.00	1.000	49.3	40.6	144.0	128.5	128.5	3.16
2	1.62	0.878	79.9	45.2	88.9	82.3	82.3	1.82
4	2.17	0.771	107.0	51.8	66.4	53.7	53.7	1.04
6	2.71	0.677	133.6	59.7	53.1	34.2	34.2	0.57
8	3.53	0.594	174.0	68.9	40.8	18.7	18.7	0.27

#### Maximum single section charge control table for different blast center distance

Based on the modified Sadofsky formula (5.2) and the comprehensive safety threshold from Table 6, we calculated the maximum single $\:H = 8\:mK = 128.6\alpha = 1.72\beta = 0.18$-section charge quantity (vertical height difference, site parameters, etc.) under varying blast center distances and corrosion depths. The calculated values were then compared with the safety factor control value of 1.5 according to current specifications, with results presented in Table 10.

**Table 10:** Control table for maximum single section charge quantity of cast iron pipelines at different burst center spacing.

Burst distance *R* (m)	Corrosion depth 0 mm	Corrosion depth: 4 mm	Corrosion depth: 6 mm	Corrosion depth: 8 mm	Safety factor 1.5 (corrosion 4 mm)	Current GB 6722 − 2014 limit value
0	12.8	2.6	1.1	0.4	1.2	0.8
5	35.7	7.2	3.1	1.1	3.4	2.2
10	92.5	18.7	8.0	2.9	8.8	5.7
15	181.3	36.6	15.7	5.7	17.3	11.2
20	301.2	60.8	26.1	9.5	28.7	18.6
25	451.7	91.2	39.1	14.2	43.0	27.9
30	632.4	127.7	54.8	19.9	60.2	39.1

Table 6 data reveals that corrosion depth is the most critical factor affecting the blasting safety threshold for cast iron pipelines, with its impact significantly exceeding that of blast source distance and internal pressure. When corrosion depth increases from 0 mm to 8 mm, the comprehensive safety threshold plummets from 128.5 cm/s to 18.7 cm/s—a 85.4% reduction—consistent with the numerical simulation finding of “253% peak stress increase at 8 mm corrosion depth.” Notably, at 4 mm corrosion depth, the safety threshold drops to 53.7 cm/s. Field measurements show a vibration velocity of 51.8 cm/s corresponding to a safety factor of merely 1.04, approaching critical failure conditions. Beyond 6 mm corrosion depth, safety factors fall below 1.0, indicating high rupture risks under standard blast loads. These findings expose fundamental flaws in current standards: The 7–15 cm/s universal limit in GB 6722 − 2014 proves overly conservative for non-corroded pipelines (with a safety factor of 3.16), severely compromising construction efficiency, while being inadequate for aging pipelines exceeding 4 mm corrosion depth. Our developed safety threshold framework employs arc-shaped corrosion defect stress concentration coefficients and material strength reduction models to enable precise risk management for pipelines with varying corrosion severity levels. Compared to traditional rectangular slot defect models, the curved defect model more accurately reflects actual corrosion morphology, avoiding excessive stress concentration coefficient calculations caused by sharp corner effects. The computational results show an error margin of less than 12% compared to field test data. This framework comprehensively considers pipe-soil coupled dynamic transfer characteristics and blast source location impacts, establishing “blast source directly beneath pipelines” as the most hazardous condition. It overturns the conventional engineering perception that “blast-facing side is critical control zone,” shifting focus to axial tensile deformation on the blast-receiving side—a conclusion corroborated by field tests demonstrating “blast-receiving side tensile strain exceeding blast-facing side by threefold.” Table 8’s single-section charge control table provides actionable operational guidelines for construction sites. Contractors need only determine maximum single-section charges based on blast center distance and pipeline corrosion detection results without complex calculations. For instance, with a 10 m blast center distance and 4 mm pipeline corrosion depth, the maximum charge capacity reaches 18.7 kg. Applying a safety factor of 1.5 reduces charges to 8.8 kg, significantly enhancing safety redundancy. Compared to current regulatory limits, the proposed charge control standards achieve substantial construction efficiency improvements while ensuring safety: for non-corroded pipelines, allowable charges reach 1.6–1.7 times regulatory limits; for pipelines with 2 mm corrosion depth, charges reach 1.3–1.4 times limits. For high-risk pipelines with corrosion depths exceeding 6 mm, protective measures such as pre-split blasting, vibration-damping trenches, and pipeline wrapping should be implemented, with vibration velocity controlled below 34.2 cm/s. When corrosion depth surpasses 8 mm, temporary pipeline relocation or reinforcement should be prioritized. The research findings in this study provide critical data support for revising the “Blasting Safety Regulations” and offer scientific evidence for safety management during blasting operations involving aging cast iron pipelines in urban renewal projects.

This paper has confirmed through full-scale tests and numerical simulations that the axial tensile strain on the protected side (the side facing the explosion) is greater and the stress concentration is more significant, making it the real weak area for controlling pipeline failure.

To quantify the differences, the conclusions of this paper are compared with those of traditional methods, the GB 6722 − 2014 specification, and typical literature results, as shown in Table 11.

**Table 11:** Quantitative comparison of the conclusions of this paper with traditional understandings, specifications and literature.

Control project	Traditional engineering understanding/literature conclusion	Current standard GB6722-2014	Experimental and simulation results of this paper	Relative difference
Control section	Forward blast surface (top/forward blast side)	Forward blast surface ground vibration	Back blast side (protected by blast)	Control positions completely different
Failure mode	Bending failure, crack on forward blast surface	No distinction of failure mode	Axial tension dominates failure	Failure mechanism different
Maximum strain location	Forward blast surface	–	Back blast side	Strain on back blast side is more than 3 times higher
Control vibration speed	7–15 cm/s	7–15 cm/s	Corrosion pipeline: 18.7–128.5 cm/s	Significant threshold difference
Most dangerous operating condition	Blast source on the side of the pipeline	Control blce	Blow source directly below the pipeline	Location of the most unfavorable position different

## Conclusion

This study, through the combination of 9 full-scale on-site blasting tests on Beijing Metro Line 16 (with a single borehole charge of 9 kg) and numerical simulation, systematically revealed the dynamic response characteristics of old gray cast iron pipes under the vibration effect of urban tunnel blasting and the corrosion-blasting coupling failure mechanism. The main conclusions are as follows:

The vibration velocity on the pipe surface increases with the decrease of the blasting center distance. The most dangerous condition occurs when the blasting source is directly below the pipe. At this time, the peak vibration velocity of the pipe reaches 40.6 cm/s, which is higher than 36.1 cm/s of the corresponding ground measurement point. The main frequency of pipe vibration is concentrated in the 10–50 Hz range, which is significantly lower than the typical frequency range of natural earthquakes. The dynamic strain of the pipe is mainly axial tensile deformation. The maximum tensile strain on the protection side of the pipe reaches 252.5 × 10⁻⁶, which is more than 3 times the minimum value (82.5 × 10⁻⁶) at the bottom of the measurement point facing the blasting side. It is clear that the protection area is the highest risk zone for tensile failure of the cast iron pipe, fundamentally overturning the traditional engineering cognition of prioritizing the protection side.

The corrosion depth is the core control factor determining the blasting safety threshold of the cast iron pipe. As the corrosion depth increases from 0 mm to 4 mm and 8 mm, the peak vibration velocity and effective stress of the pipe significantly increase. At 8 mm corrosion depth, the peak effective stress of the pipe is 253% higher than that in the non-corroded state, and the stress peak and vibration peak occur simultaneously at 0.03 s after the initiation. Strong stress concentration occurs at the corrosion defect, causing the vibration velocity of the defect area to exhibit irregular “W” shape fluctuations. The stress extremes always concentrate at the central cross-section of the corrosion defect. The blasting vibration disrupts the static pressure balance inside the pipe, further exacerbating the damage in the corrosion area with high stress, significantly increasing the risk of sudden pipe rupture.

The current “Blasting Safety Regulations” (GB 6722 − 2014) do not consider the corrosion damage characteristics of cast iron pipes. The vibration velocity safety threshold used in domestic and foreign engineering practices varies greatly (2–25 cm/s), resulting in a dilemma of “conservative control delaying the project schedule and loose control triggering accidents” in engineering applications. The corrosion-blasting coupling response theory and arc-shaped defect numerical model established in this study clarified the safety threshold corresponding to different corrosion depths (the safety threshold decreases by 85.4% at 8 mm corrosion depth), and the proposed single-bore charge control table can be directly applied to engineering practice, providing key scientific basis for the revision of blasting safety standards and the optimization of construction parameters for old cast iron pipes.

This study still has certain limitations: The on-site tests were conducted only on gray cast iron pipes of specific diameters and wall thicknesses, and did not cover other commonly used pipe materials such as ductile iron, nor did they cover pipes with different structural parameters; only uniform arc-shaped corrosion defects were considered, and more complex corrosion forms such as pitting, cracks, and weld damage were not involved; the test conditions were limited to a single hole with a 9 kg charge quantity, and the influence of blasting parameters such as charge quantity, initiation sequence, and hole layout was not systematically studied; at the same time, the coupling effects of environmental factors such as pipe internal pressure, soil properties and thickness, and surrounding structures were not fully considered. Further research will expand the scope of research conditions and parameters, establish a multi-factor coupled pipeline blasting safety assessment system, and provide more comprehensive technical support for the full life cycle safety protection of urban underground pipelines.

## Data Availability

The data in this study are presented in the full manuscript.
